# Editorial: Charophytes: Evolutionary Ancestors of Plants and Emerging Models for Plant Research

**DOI:** 10.3389/fpls.2017.00338

**Published:** 2017-03-14

**Authors:** David Domozych, Iben Sørensen, Zoë A. Popper

**Affiliations:** ^1^Department of Biology, Skidmore Microscopy Imaging Center, Skidmore CollegeNew York, NY, USA; ^2^Plant Biology Section, School of Integrative Plant Sciences, Cornell UniversityIthaca, NY, USA; ^3^Botany and Plant Sciences, School of Natural Science, National University of IrelandGalway, Ireland

**Keywords:** charophytes, model, evolution, streptophytes, algae

Approximately 450–500 million years ago, an ancient freshwater green alga successfully colonized land. From this profoundly important event in our planet's natural history, the spectacular diversity of plants that now occupy most of our terrestrial ecosystems arose. Essentially, the planet's atmosphere and biogeochemistry dramatically changed to yield conditions that support our biota. Land plant evolution also directed the origin and development of human civilization as it ultimately served as the basis of agriculture, clothing, building and medicines, to name just a few. The Charophycean Green Algae or charophytes (also known as streptophyte algae) are *the* group of green algae that are ancestral to land plants. Extant charophyte taxa also share a large number of features that are found in modern land plants. Over the past half century, studies of charophytes have been inspired by this evolutionary significance. Yet as we have learned more about the intricacies of their biology, charophyte research has notably expanded and several inclusive taxa have become important “tools” in understanding the foundations of plant life. This has been further complemented by many advantageous experimental attributes of charophytes including their small size, their efficacious accommodation to molecular and high resolution imaging technologies and their relative ease in experimental manipulation. Many, in fact, have become or are becoming important model organisms in several areas of basic plant biology (Domozych et al.). The Frontiers in Plant Science series, “Charophytes: Evolutionary ancestors of plants and emerging models for plant research” provides a collection of reports and reviews that demonstrate the ever growing importance of charophytes in plant research.

One of most well-known and studied charophytes is the unicellular desmid, *Micrasterias*, whose spectacular morphology and associated cellular morphogenesis make it ideal for elucidating the subcellular foundations of cell development and physiology. In this series on charophytes, Lütz-Meindl provides a detailed summary of past and current research efforts that have focused on *Micrasterias*. Though cell expansion, subcellular dynamics and cell wall development have been important focal points of previous study, this alga is now benefitting ecophysiological analyses especially in the elucidation of adaptations to various stresses. Other desmids as well have contributed to foundational areas of biological research. For example, species of *Closterium* provide excellent models for studying reproductive biology. In this series, Abe et al. demonstrate this using lectin cytochemistry and electron microscopy to describe putative molecules required for gamete release during sexual reproduction.

Desmids are not sole “stars” of charophyte-based research. The stoneworts (e.g., *Chara* and *Nitella*) have long been important to plant research. These algae produce exceptionally large internodal cells that are uniquely valuable to various cellular studies. Belby describes the distinct features of these algae and specifically reports on the diverse methodologies that have been used and key results that have been obtained in studies dealing with electrophysiology, auxin dynamics and membrane transporters. Central to the physiology of any plant cell including *Chara* is endomembrane dynamics especially the coordinated balance between exo- and endocytosis in the membrane trafficking network. Foissner et al. describe membrane dynamics in the internodal cells of *Chara* when treated with the fungal metabolite, wortmannin. Using immunofluorescence and transmission electron microscopy-based imaging they demonstrate significant reorganization of the trans Golgi network (TGN) and changes to the unique membranous charasomes. Ultimately, the expansion and development of the cells of *Chara*, as in all plants, is controlled by turgor and the structural dynamics of the cell wall. Pectins represent the largest domain of the wall matrix polysaccharides that are critical to cell development. Boyer elegantly describes the distinctive pectin-Ca^2+^ cycle in the *Chara* cell wall that allows for controlled wall expansion and possible implications for plant cells in general.

Charophytes are now also becoming important organism in studies focused on stress-induced adaptations of plant cells. These studies are not only important in understanding survival mechanisms of plants under various stresses but also provide insight into the evolutionary processes that may have led to colonization of land by ancient charophytes. Holzinger and Pichrtova explore various adaptive mechanisms including biochemical, structural and physiological processes that extant charophytes employ for survival on land. Additionally, they describe the molecular machinery that modulates when these algae are under particular pressures such as exposure to UV light and desiccation. In the same vein, Kondo et al. used genomic sequence analysis and biochemistry to identify a lipid-like aliphatic component in the early divergent charophyte, *Klebsormidum*. The authors discuss the existence of cutin-like substances that may function under stress in a similar way to the cuticular waxes of land plants.

The study of charophytes is still in an early but exciting phase of discovery with much yet to be resolved. Areas that will require extensive research efforts are the molecular phylogeny and taxonomy of the diverse taxa within this algal group. Lemieux et al. employ chloroplast genome analyses to decipher major structural alterations amongst three groups of charophytes, specifically focusing on the large inverted repeat sequence encoding the rRNA operon.

This series on charophytes serves as only a brief sampling of the intriguing biology of these algae. It is hoped that this will encourage and enthuse many more plant scientists to incorporate these algae in their research.

## Author contributions

All authors listed, have made substantial, direct and intellectual contribution to the work, and approved it for publication.

## Funding

This work was funded by NSF-MCB grant 1517345.

### Conflict of interest statement

The authors declare that the research was conducted in the absence of any commercial or financial relationships that could be construed as a potential conflict of interest.

